# Datopotamab deruxtecan in breast cancer treatment: a systematic review of clinical efficacy and safety

**DOI:** 10.3389/fimmu.2026.1899080

**Published:** 2026-08-27

**Authors:** Julia Piekarz, Natalia Picheta, Jakub Pobideł, Natalia Gierulska, Krzysztof Kułak, Rafał Tarkowski

**Affiliations:** 1 Student’s Scientific Association—I Chair and Department of Gynecological Oncology and Gynecology, Medical University of Lublin, Lublin, Poland; 2 I Chair and Department of Gynecological Oncology and Gynecology, Medical University of Lublin, Lublin, Poland

**Keywords:** datopotamab deruxtecan, antibody–drug conjugate, Trop-2, breast cancer, triple-negative breast cancer, tumor microenvironment, immunogenic cell death

## Abstract

**Background:**

Breast cancer (BC) remains the most common malignant tumor in women worldwide and a leading cause of cancer-related deaths. Despite therapeutic advances, patients with advanced BC, particularly triple-negative breast cancer (TNBC), still face limited effective treatment options. Antibody-drug conjugates (ADCs) represent a compelling strategy that combines precise tumor targeting with cytotoxic payloads, effectively bridging targeted delivery with microenvironmental disruption. Datopotamab deruxtecan (Dato-DXd), a Trop-2-targeted ADC, has demonstrated encouraging clinical activity. Preclinical evidence suggests that Dato-DXd may modulate the tumor microenvironment (TME) through potential induction of immunogenic cell death (ICD), providing a biological rationale for investigating synergistic combinations with immunotherapy.

**Materials and methods:**

A systematic review was conducted in accordance with PRISMA guidelines using the PubMed, Web of Science, and ClinicalTrials databases. The inclusion criteria targeted relevant clinical studies published between 2024 and 2026. Ultimately, after a thorough analysis, one randomized controlled trial (RCT) and one single-arm study met the eligibility criteria and were included in the review.

**Results:**

Data analysis from the Phase III TROPION-Breast01 trial demonstrated that Dato-DXd significantly improved objective response rate (ORR) and median progression-free survival (PFS) compared to investigator’s choice chemotherapy in patients with HR+/HER2- breast cancer. In the Phase I TROPION-PanTumor01 trial, Dato-DXd showed promising clinical activity in both HR+/HER2- breast cancer and TNBC cohorts. Regarding safety, Dato-DXd was associated with a lower incidence of grade ≥ 3 adverse events compared to standard chemotherapy in the randomized setting, although low-grade toxicities such as stomatitis and nausea remained common.

**Conclusion:**

Dato-DXd demonstrates robust clinical activity in patients with BC, providing superior disease control with a favorable safety profile compared to conventional chemotherapy. Beyond direct tumor cell ablation, its mechanism of action includes a potent bystander killing effect, while preclinical data suggest that the induction of ICD may contribute to reshaping a heterogeneous TME. Future research is needed to correlate these preclinical mechanistic hypotheses with clinical outcomes. The limited number of available RCTs and Real-World Evidence (RWE) studies underscores the need to raise awareness and conduct further research to identify predictive biomarkers and evaluate combinations with immune checkpoint inhibitors to optimize personalized treatment regimens.

**Systematic review registration:**

https://www.crd.york.ac.uk/prospero/, identifier CRD420261408796.

## 1 Introduction

Breast cancer (BC) is one of the most common cancers affecting women worldwide, with around 80% of cases occurring in women over the age of 50. Due to its high incidence and mortality rates, it has now become one of the major health concerns (1). According to the latest statistics published in 2024 – GLOBOCAN 2022, based on data from 185 countries worldwide, BC is the second most common cancer, with as many as 2,308,897 new cases detected. The highest incidence rates are observed in France. Furthermore, in Australia and New Zealand, North America and Northern Europe, incidence rates are four times higher than in South-Central Asia and Central Africa. In terms of mortality, BC ranks fourth with 665,684 deaths in 2022, with the highest number of deaths recorded in Melanesia (2).

Molecular analysis of BC combined with gene expression profiling allows BC to be classified into different subtypes: estrogen receptor (ER)-positive luminal – luminal A and luminal B, Human Epidermal Growth Factor Receptor (HER2)-enriched and basal. A detailed description of the types of BC is presented in **Table 1** (3).

**Table 1:** Comparison of molecular types of BC (3). Triple-negative breast cancer (TNBC), progesterone receptor (PR).

Molecular subtype	Receptor status	Characteristics	Prognosis
Luminal A	ER+, PR+, HER2-, low Ki-67	The most common, grows slowly, not very aggressive	The best outcome, with the lowest risk of local recurrence and metastasis.
Luminal B	ER+, PR+/-, HER2+/-, high Ki-67	Higher proliferative activity than in type A.	Good/Moderate; higher risk of recurrence than in type A.
HER2-positive	ER-, PR-, HER2+	Highly aggressive, rapid growth, lymph nodes often affected.	Thanks to modern targeted drugs, prognoses have improved significantly.
TNBC	ER-, PR-, HER2-	The most aggressive, often associated with a BRCA1 mutation.	The worst-case scenario: a high risk of early recurrence – particularly within the first 3–5 years.

Antibody-drug conjugates (ADCs) currently offer new hope for BC patients. ADCs consist of monoclonal antibodies (mAbs), a stable linker and a potent cytotoxic agent. mAbs play a key role in the selective recognition of and binding to antigens specific to cancer cells. Their structure is based on that of immunoglobulin, most commonly IgG1. It acts as a selective carrier, enabling the distinction between cancer cells and healthy tissue – primarily via the antigen (Fab). For mAbs to be effective, they must have sufficient affinity for the target antigen, minimal or no binding to other receptors, and the ability to penetrate cancer cells. Furthermore, they must have low immunogenicity, a long half-life and high stability. The crystallized fragment (Fc) is responsible for extending the half-life; by integrating with the Fc receptor (FcRn), it prevents premature degradation. Another structural component of ADCs are linkers. These are divided into two groups: degradable and non-degradable. They allow cytotoxic agents to be linked to monoclonal antibodies, thereby ensuring stability during systemic circulation. This is intended to minimize premature drug release, reduce off-target toxicity and mitigate immune reactions. Furthermore, the linker must not induce ADC aggregation, as this could lead to antibody malfunction, reduced stability and altered pharmacokinetics. At the same time, linkers play a regulatory role in the release of cytotoxic agents within target cells. Only after the complex has entered the cell is the cytotoxic agent released. This leads to the death of the cancer cell, whilst preserving healthy tissues (4, 5).

Beyond traditional targeted cytotoxicity, modern ADCs are hypothesized to interface with and potentially reshape the tumor microenvironment (TME). In preclinical studies, the engagement of the antibody’s crystallized fragment (Fc) with immune effector cells has been shown to trigger antibody-dependent cellular cytotoxicity (ADCC) or complement-dependent cytotoxicity (CDC) (6). Furthermore, preclinical models suggest that payload delivery may induce immunogenic cell death (ICD), prompting the release of damage-associated molecular patterns (DAMPs) into the TME. This immunogenic cascade has been proposed to facilitate the conversion of immunologically ‘cold’ tumors into ‘hot’ tumors by stimulating dendritic cell maturation and boosting tumor-infiltrating lymphocyte (TIL) recruitment, thereby offering a mechanistic rationale for combining ADCs with contemporary immune checkpoint inhibition. However, these mechanisms remain primarily characterized in experimental settings, and their clinical impact in breast cancer patients requires further validation (7).

Modern methods of BC treatment are tailored to patients in various respects. The choice of treatment pathway is strictly dependent on tumor biology, the assessment of recurrence risk, and the patient’s general condition. According to the latest St. Gallen 2025 and European Society for Medical Oncology (ESMO) guidelines, hormone therapy forms the cornerstone of treatment for the HR+/HER2- hormonal subtype. Tamoxifen is most commonly used in premenopausal women, while ovarian suppression is recommended for patients at higher risk. For women with BC susceptibility gene 1/2 (BRCA1/2) mutations who are at high risk of recurrence, the current standard of care also includes the administration of olaparib. In the case of HER2-positive and triple-negative breast cancers (TNBC), the preferred approach—particularly for tumors larger than 2 cm or those with lymph node involvement—is neoadjuvant (preoperative) treatment. In the HER2+ subtype, the standard of care is chemotherapy combined with dual anti-HER2 blockade – trastuzumab and pertuzumab. In contrast, for patients in whom residual disease is detected post-surgery, a switch to the trastuzumab emtansine (T-DM1) conjugate is necessary. A breakthrough in the treatment of TNBC was the addition of pembrolizumab immunotherapy to neoadjuvant chemotherapy. This led to an increase in the rate of complete pathological responses and improved overall survival (OS) (8).

A new ADC currently in the research phase is datopotamab deruxtecan (Dato-DXd). It is a drug targeting the trophoblast cell-surface antigen 2 (Trop-2) glycoprotein. Trop-2 plays a significant role in breast cancer pathology by acting as a transmembrane calcium signal transducer that promotes cancer cell proliferation, migration, and survival. Its overexpression is frequently observed across various breast cancer subtypes and is generally associated with increased tumor aggressiveness and a poor clinical prognosis. Based on the TROPION-Breast01 trial, a phase III study, a statistical and clinical improvement in progression-free survival (PFS) was demonstrated in patients with previously treated, inoperable or metastatic HR+/HER2- BC compared to standard chemotherapy (9).

The aim of this paper is to present the current state of knowledge regarding Dato-DXd and to evaluate its potential role in the treatment of BC, with particular emphasis on patients with advanced or difficult-to-treat disease. Given the limited efficacy of standard therapeutic approaches and the ongoing need for more effective and less toxic treatment strategies, Dato-DXd appears to be a favorable therapeutic option. The analysis of currently available clinical studies on the efficacy and safety profile of this agent may contribute to a better understanding of its significance in modern BC therapy, as well as indicate directions for further research and potential clinical applications.

## 2 Materials and methods

### 2.1 Study designs

This systematic review was conducted in accordance with the Preferred Reporting Items for Systematic Reviews and Meta-Analyses (PRISMA) 2020 guidelines. The aim of the review was to comprehensively assess the efficacy and safety of Dato-DXd in the treatment of HR+/HER2- BC or TNBC. The analysis included different types of studies: randomized controlled trials (RCT) and single-arm studies, to provide a broad perspective on available evidence. Because Dato-DXd has only recently been introduced into clinical trials, the number of available studies meeting the inclusion criteria was limited.

The review protocol was registered in the International Prospective Register of Systematic Reviews (PROSPERO) under registration number CRD420261408796.

### 2.2 Eligibility criteria and PICO framework

The research focused on evaluating the efficacy and safety of the therapy. The PICO model was used to structure and focus the literature review.

- Population: The target population consists of adult patients suffering from HR+/HER2- BC or TNBC, regardless of gender.
- Intervention: interventions include the use of Dato-DXd monotherapy.
- Comparison: A comparative analysis of Dato-DXd therapy versus chemotherapy was conducted. For single-arm studies, results were analysed without comparison with a control group.
- Outcome: The results analyzed in the review focused on evaluating the efficacy and safety of Dato-DXd therapy in patients with HR+/HER2- BC or TNBC. Efficacy was primarily assessed based on objective response rate (ORR), PFS, OS, duration of response (DoR), and other clinical parameters when available. The safety of the therapy was analyzed by assessing the incidence of treatment-related adverse events (TRAEs), including serious adverse events (≥ Grade 3).

Studies published in English between 2024 and 2026 were included in the analysis. RCT and single-arm studies were included if they contained data on the use of Dato-DXd in the patient population in question. Exclusion criteria included review publications, reports of preclinical studies (both in animal and cellular models), abstracts without complete data (e.g., conference abstracts or posters that did not provide full results), and papers that did not include information on the efficacy or safety of Dato-DXd treatment.

A summary of the inclusion and exclusion criteria based on the PICO framework is presented in **Table 2**.

**Table 2:** Eligibility criteria based on the PICO framework.

Component	Criteria
Population (P)	Adult patients diagnosed with HR+/HER2-BC or TNBC, any gender
Intervention (I)	Datopotamab deruxtecan (Dato-DXd) monotherapy
Comparison (C)	Standard chemotherapy or no comparator
Outcomes (O)	Efficacy: ORR, CR, PR, SD, PD, PFS, OS, DoR; Safety: TRAEs
Study Design	RCT, Single arm studies
Publication years	2024–2026
Language	English.
Exclusion criteria	Review publications, preclinical studies (animal or cellular models), abstracts or conference posters without complete data, studies not reporting efficacy or safety outcomes of Dato-DXd treatment

### 2.3 Search and selection process

A systematic search of PubMed, Web of Science, and ClinicalTrials.gov was performed to identify relevant studies published between January 1, 2024, and February 28, 2026. The final search was conducted on May 1, 2026. Keywords used included: “Datopotamab Deruxtecan”; “Triple-negative breast cancer”; “Antibody–drug conjugate”; “Breast cancer” and “Trop-2”. To ensure the completeness of the review, a manual search of the results and an evaluation of the bibliographies of the selected articles was also conducted. Two researchers (JuP and NP) independently searched the databases and critically appraised the selected articles. Any discrepancies were resolved by verification by two other authors (NG and JaP).

**Figure 1:**
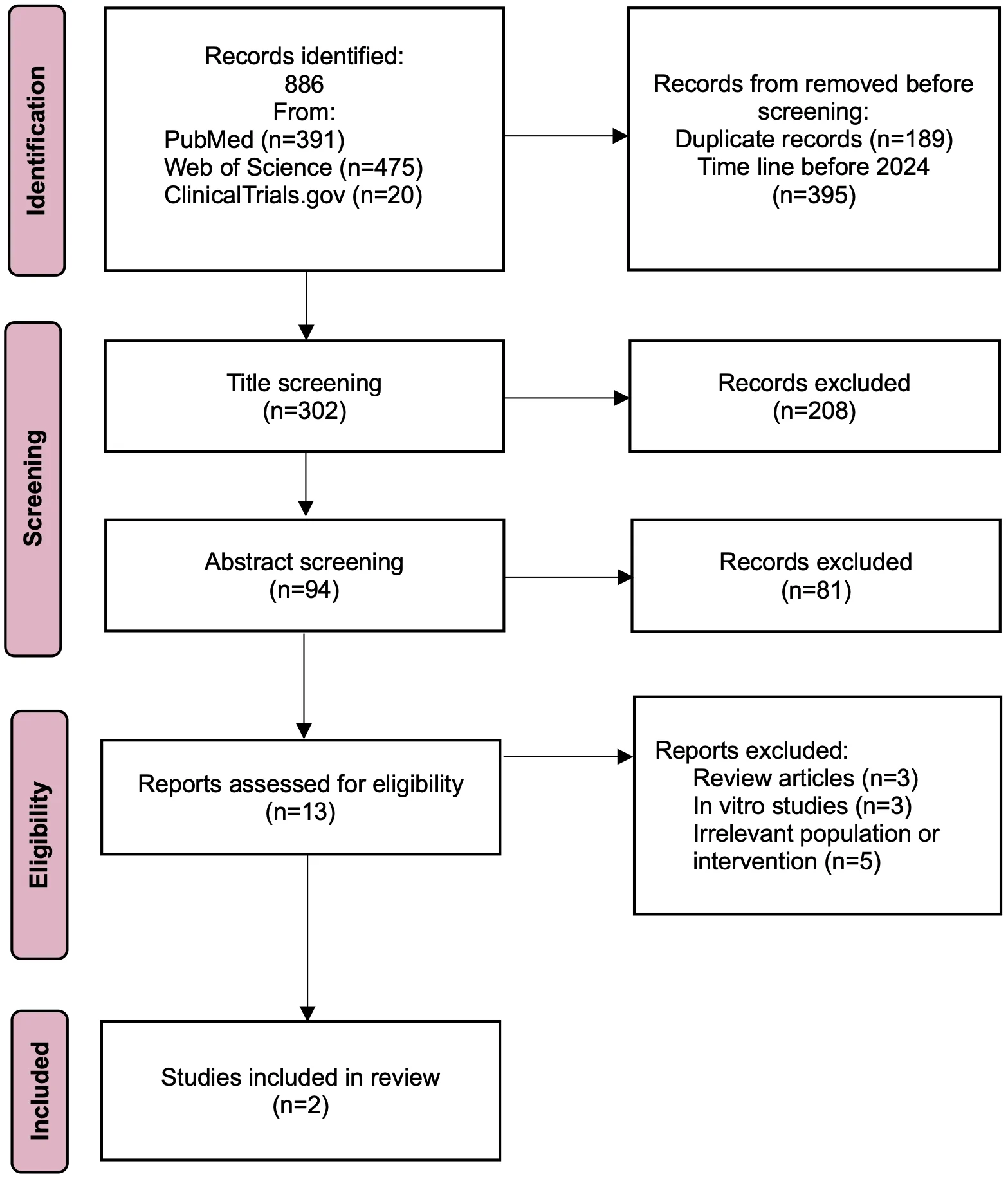
Preferred reporting items for systematic reviews and meta-analyses (PRISMA) flow diagram of study identification, inclusion, and exclusion.

A systematic database search identified 886 records, of which, 395 records were excluded for not meeting the temporal inclusion criteria, and 289 records were omitted due to irrelevance of outcomes or results. An additional 11 records were excluded as they comprised review articles, *in vitro* studies and irrelevant population or intervention. Ultimately, 2 articles met the inclusion criteria and were incorporated into the primary analysis. Following a quality assessment, all 2 studies were deemed to be of high methodological quality. The comprehensive data selection and identification process is illustrated in **Figure 1**.

### 2.4 Data extraction

The review included one RCT and one single-arm study involving a total of 817 participants diagnosed with HR+/HER2- BC or TNBC and treated with Dato-DXd. Data extraction was performed independently by two reviewers using a standardized data extraction form. Extracted data included study characteristics (author, year, study design), patient demographics (total number of patients, age, gender), intervention details (Dato-DXd dosage, regimen), and outcome measures related to efficacy (ORR, PFS, OS, DoR, Complete Response (CR), Partial Response (PR), Stable Disease (SD), Progressive Disease (PD), Clinical Benefit (CB) and safety (incidence and severity of TRAEs). Any discrepancies were resolved through discussion or consultation with a third reviewer.

### 2.5 Assessment of risk of bias in the included studies

The included studies comprised RCT and single-arm studies.

**Figure 2:**
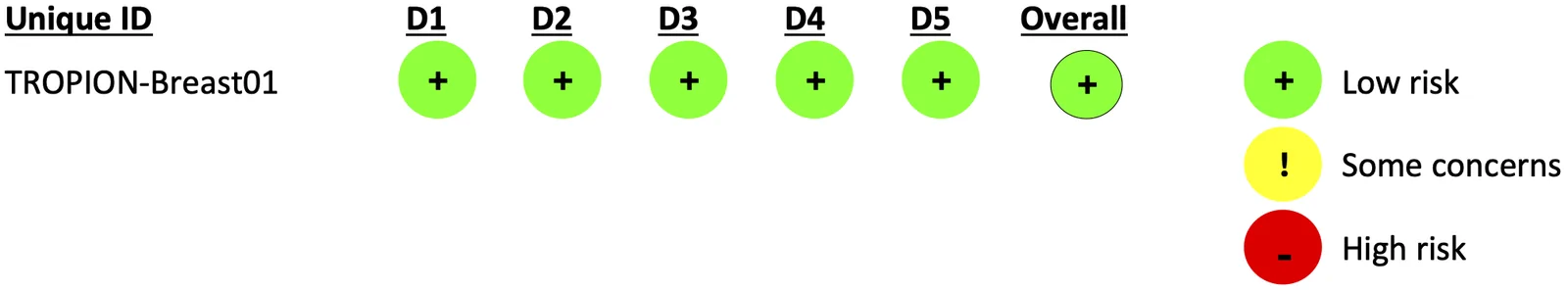
Risk of bias assessment for the included randomized controlled trial (TROPION- Breast01) using the Cochrane Risk of Bias 2.0 (RoB 2.0) tool. Risk of bias is evaluated across five specific domains: D1: Bias arising from the randomisation process; D2: Bias due to deviations from the intended interventions; D3: Bias due to missing outcome data; D4: Bias in measurement of the outcome; and D5: Bias in selection of the reported result. The green circles with a plus sign (+) indicate a low risk of bias.

For the RCT, risk of bias was assessed using the updated Cochrane Risk of Bias tool for randomized trials (RoB 2.0) (Higgins, 2019). This tool evaluates five domains: (1) bias arising from the randomization process, (2) bias due to deviations from intended interventions, (3) bias related to missing outcome data, (4) bias in outcome measurement, and (5) bias in the selection of reported results. Two independent reviewers applied RoB 2.0 to the all included RCT, recording supporting evidence and providing judgments of low risk, high risk, or some concerns for each domain. Discrepancies were resolved by discussion or adjudication by a third reviewer. The detailed risk of bias assessment for the RCT according to the Cochrane RoB 2.0 tool is illustrated in **Figure 2**.

For the single-arm studies, the methodological quality was textually evaluated using the NIH Quality Assessment Tool for Before-After (Pre-Post) Studies with No Control Group. The included study [TROPION-PanTumor01] demonstrated high methodological rigor, successfully fulfilling 10 out of the 11 applicable criteria (with one criterion deemed not applicable). The single domain where the study design fell short was the blinding of outcome assessors, which is inherent to the open-label nature of early-phase oncology trials. Consequently, the study was graded as having an overall quality rating of “Good”, indicating a low risk of bias and high reliability of the reported clinical outcomes.

This tailored approach to risk of bias assessment, specific to each study design, ensured a comprehensive evaluation of study quality and enhanced the overall reliability of the review findings.

## 3 Datopotamab deruxtecan

Dato-DXd is an advanced ADC optimized for precise targeting of the transmembrane glycoprotein Trop-2 (encoded by the TACSTD2 gene). The Dato-DXd molecule architecture is based on a humanized IgG1 monoclonal antibody (datopotamab), which is covalently linked to a potent topoisomerase I inhibitor (an exatecan derivative, DXd) via a tetrapeptide linker (Gly-Gly -Phe-Gly) (10). Unlike previous generations of ADCs, the molecular engineering of Dato-DXd involved a deliberate reduction of the drug-to-antibody ratio (DAR) to ~4. Such optimization is crucial for balancing cytotoxic potential with the pharmacokinetic stability of the molecule in systemic circulation, minimizing premature release of the drug (11).

**Figure 3:**
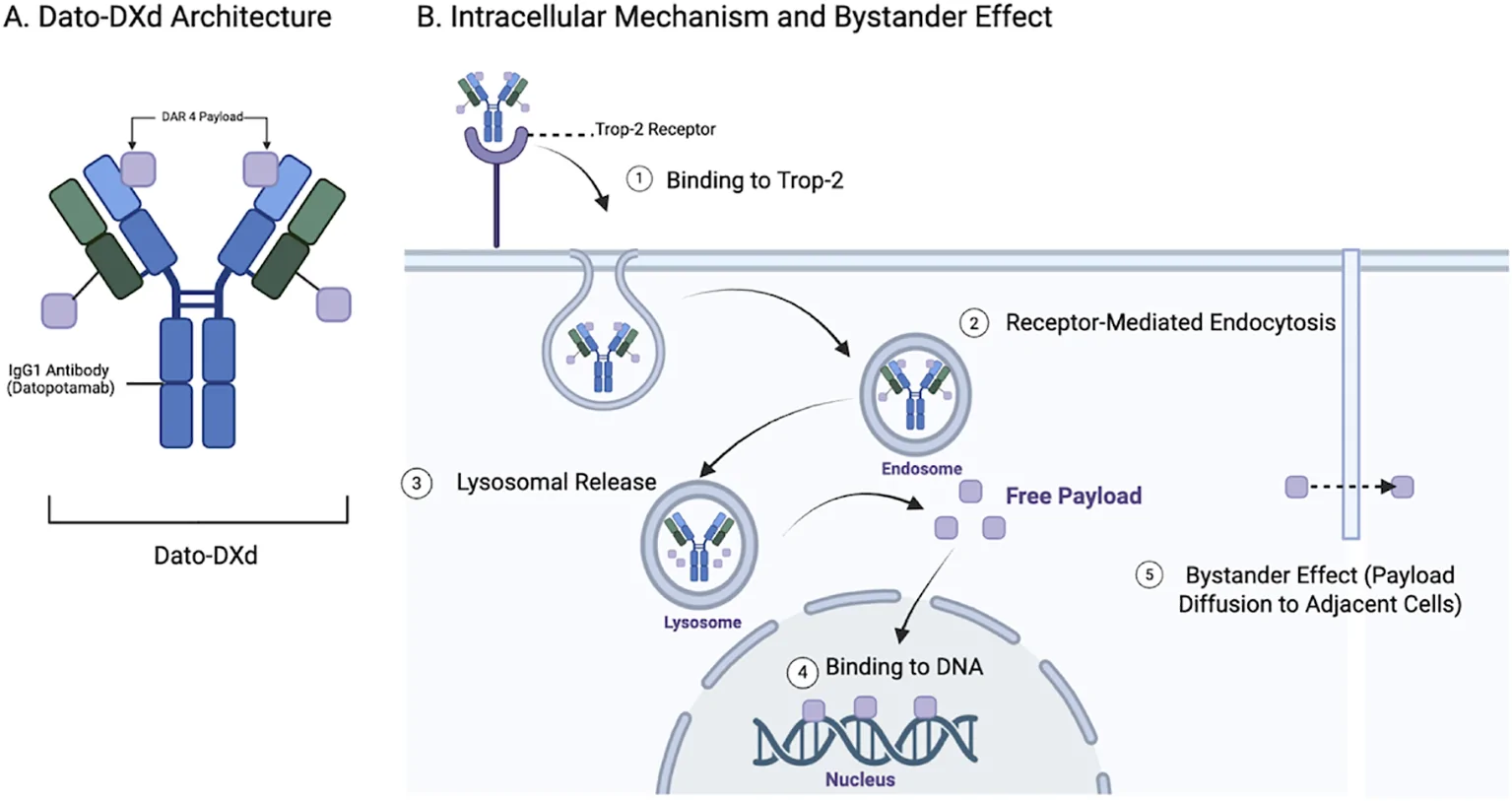
Structural architecture and mechanism of action of Dato-DXd. **(A)** Molecular structure of Dato-DXd. **(B)** Intracellular trafficking: Trop-2 binding, endocytosis, lysosomal degradation, and topoisomerase I inhibitor release leading to DNA damage. The scheme also illustrates the bystander killing effect and hypothesized ICD via DAMP release.

From a biopharmaceutical perspective, the maleimide-based linker in Dato-DXd exhibits exceptional stability in plasma—only about 5% of the DXd payload is released into the circulation over a 21-day period (10). As illustrated in **Figure 3**, after specific binding to the Trop-2 receptor on the surface of the cancer cell, the ADC complex is internalized via receptor-mediated endocytosis. Within the acidic environment of lysosomes, the linker is specifically cleaved by endogenous proteases, including cathepsin B, releasing the cytotoxic deruxtecan directly into the cytoplasm. DXd penetrates into the cell nucleus, where it stabilizes topoisomerase I-DNA cleavage complexes, inducing irreversible breaks in both strands of the nucleic acid and, consequently, cell death via apoptosis. Due to deruxtecan’s high lipophilicity and permeability through cell membranes, Dato-DXd is thought to exert a highly effective bystander killing effect. The charged molecules are hypothesized to penetrate into neighboring cancer cells lacking Trop-2 expression, which may allow for overcoming tumor antigenic heterogeneity. Crucially, as shown in **Figure 3**, preclinical models suggest that this cellular destruction triggers immunogenic cell death (ICD), releasing vital damage-associated molecular patterns (DAMPs)—such as HMGB1 and extracellular ATP—into the extracellular space. These DAMPs are believed to act as potent ‘find me’ and ‘eat me’ signals that actively recruit, activate, and promote the maturation of antigen-presenting cells, particularly dendritic cells (DCs), within the tumor microenvironment. By turning the baseline apoptotic process into a coordinated trigger for localized microenvironmental activation, it is postulated that this biological cascade effectively bridges targeted payload cytotoxicity with the initiation of a robust adaptive immune response (12).

The structural and pharmacodynamic differences of the anti-Trop-2 class become evident when compared to other ADCs used in BC. Sacituzumab govitecan (SG) utilizes an anti-Trop-2 antibody (hRS7) conjugated to the SN-38 payload via a hydrolyzable linker (CL2A) with a higher DAR of 7.6. The significant instability of the SG linker in plasma (release of ~90% of the payload within 3 days) determines a toxicity profile dominated by severe hematologic (e.g., neutropenia) and gastrointestinal (e.g., diarrhea) events (13). In the case of Dato-DXd, the high stability of the linker and the optimized payload result in a shift in the adverse event profile toward stomatitis and ocular complications, arising directly from the physiological expression of Trop-2 on normal mucosal and corneal epithelia (14).

In contrast, when compared to trastuzumab deruxtecan (T-DXd, HER2-targeted), which shares the same payload and release mechanism with Dato-DXd, Dato-DXd differs in having a lower DAR (4 vs. ~8) (15). The decision to lower the DAR for Dato-DXd was based on the widespread distribution of Trop-2 in normal body tissues. This allowed for a broader therapeutic window for the drug and reduced the incidence of severe pulmonary events, such as interstitial lung disease (ILD), which occurs much less frequently with Dato -DXd. However, sharing the identical topoisomerase I (TOP1) inhibitor moiety carries a risk of partial cross-resistance when T-DXd and Dato-DXd are used sequentially. This results from selective pressure promoting acquired mutations in DNA repair pathway genes (e.g., SLX4 gene mutations) or conformational changes in the TOP1 enzyme itself (e.g., S57C, R364H mutations) (10).

In a translational context, the role of Trop-2 as a predictive biomarker defies the classic paradigm observed for HER2. Although widespread Trop-2 overexpression in BC (particularly in the triple-negative and luminal subtypes) is associated with a poorer prognosis, the correlation between the degree of its membrane expression and the objective response to Dato-DXd treatment is not strictly linear. The presence of Trop-2 is an indisputable prerequisite for internalization, but analyses of circulating tumor cells (CTCs) demonstrate that acquired clinical resistance to Dato-DXd rarely results from evolutionary down-regulation of the target antigen itself. Clinical responses are observed even in patients with low Trop-2 expression, which is partly explained by a strong proximity effect (16). This complicates the implementation of simple eligibility criteria, prompting the development of standardized pathomorphological algorithms—such as the Quantitative Continuous Scoring (QCS) system, which assesses a normalized membrane ratio to more precisely identify subpopulations that benefit most from therapy. The primary drivers of loss of response remain adaptive intracellular modifications in DNA damage response (DDR) pathways and tumor stroma remodeling—e.g., activation of the cancer-associated fibroblast (CAF)-focal adhesion kinase (FAK) signaling axis. This stromal activation creates a dense extracellular matrix that physically impedes the deep penetration of large ADC macromolecules into the core of the TME, creating a spatial barrier to treatment efficacy (17).

The biological efficacy of the Dato-DXd mechanism has translated into tangible benefits in clinical oncology, as confirmed by the results of the Phase III TROPION-Breast01 trial. Dato-DXd demonstrated a statistically significant advantage over chemotherapy in terms of prolonging progression-free survival (PFS) and achieving a higher objective response rate (ORR) (18). A direct result of this translational success was the decision by the U.S. Food and Drug Administration (FDA), which in January 2025 approved the drug Datroway (datopotamab deruxtecan-dlnk) for the treatment of adult patients with unresectable or metastatic HR+/HER2- BC, following prior hormone therapy and systemic chemotherapy. Additionally, the drug was approved by the European Medicines Agency (EMA) in the same year (19, 20).

Furthermore, the highly specific mechanism of Dato-DXd demonstrates broad potential within epithelial tumors. Robust evidence of anticancer activity obtained in the TROPION-Lung01 study in patients with advanced non-small cell lung cancer (NSCLC) positions this conjugate as a breakthrough tool in combating resistant tumors with a high proliferation rate and abundant Trop-2 expression (21).

## 4 Clinical trials

### 4.1 TROPION-PanTumor01 (NCT03401385)

The TROPION-PanTumor01 study was an open-label, multicenter Phase I clinical trial evaluating the safety and efficacy of Dato-DXd in patients with advanced solid tumors. The primary objective of the study was to evaluate the safety and tolerability of the drug, while secondary endpoints included antitumor activity and pharmacokinetics. The study enrolled 85 patients aged ≥18 years with diagnosed BC, including HR+/HER2– BC (n=41) or TNBC (n=44), who had experienced disease progression or recurrence following standard therapy. Participants received Dato-DXd at a dose of 6–8 mg/kg intravenously every 3 weeks.

An assessment of drug tolerance and safety showed that all patients experienced some form of treatment-related adverse events (TRAEs), while grade 3 or higher TRAEs were observed in 41.5% of patients with HR+/HER2– BC and 52.3% of patients with TNBC. The most commonly observed TRAE was stomatitis, which was observed in 80.5% of patients with HR+/HER2- BC and 72.7% of those with TNBC. Other frequently reported TRAEs included nausea, fatigue, hematologic abnormalities, dry eye, rash, and skin hyperpigmentation. The detailed incidence of all TRAEs with Dato-DXd therapy is presented in **Table 3**. Most events were mild or moderate in nature, while severe, life-threatening, and fatal events occurred much less frequently (18).

**Table 3:** Incidence of TRAEs in patients with HR+/HER2- BC and TNBC receiving Dato-DXd (18).

TRAEs	HR+/HER2- BC (n=41)		TNBC (n=44)	
	Any TRAEs	Grade ≥3	Any TRAEs	Grade ≥3
Stomatitis	80.5%	9.8%	72.7%	11.4%
Nausea	51.2%	0%	65.9%	2.3%
Fatigue	43.9%	0%	34.1%	6.8%
Alopecia	36.6%	0%	36.4%	0%
Dry eye	22%	0%	11.4%	0%
Dry mouth	9.8%	0%	6.8%	0%
Headache	12.2%	0%	15.9%	0%
Vomiting	22%	0%	29.5%	4.5%
Diarrhea	17.1%	0%	9.1%	0%
Anemia	7.3%	4.9%	11.4%	2.3%
Lymphopenia	2.4%	2.4%	9.1%	0%
Skin hyperpigmentation	12.2%	0%	4.5%	0%
Rash	14.6%	0%	13.6%	0%
Decreased appetite	12.2%	0%	15.9%	0%

An analysis of the efficacy of Dato-DXd showed that the ORR in patients with HR+/HER2- BC was 26.8% (95% CI: 14.2–42.9), and in patients with TNBC, it was 31.8% (95% CI: 18.6–47.6). PRs accounted for the majority of observed responses (26.8% and 29.5%), while CRs were rare (0% and 2.3%). Another evaluated endpoint was median DoR—it was not reached (NE) in the HR+/HER2- BC subtype group, and in the TNBC group it was 16.8 months. SD was observed in 56.1% and 40.9%, while PD was observed in 12.2% and 18.2%, respectively. In terms of survival parameters, the median PFS in patients with HR+/HER2- BC was 8.3 months, whereas in the TNBC group, the median PFS was documented at 4.4 months, fully aligning with the baseline therapeutic expectations for pretreated aggressive disease. The median OS was 13.5 months in TNBC, while in the HR+/HER2- subtype it was NE. DCR was also assessed, which was 85.4% in the HR+/HER2- BC group and 79.5% in the TNBC group (18).

The results of the TROPION-PanTumor01 study indicate significant antitumor activity of Dato-DXd in patients with advanced BC, with a predictable and manageable safety profile. The observed responses demonstrated potential durability, underscoring the clinical significance of this therapy in previously heavily pretreated populations where the tumor microenvironment is often characterized by advanced immunosuppression and profound clonal heterogeneity.

### 4.2 TROPION-Breast01 (NCT05104866)

The TROPION-Breast01 trial was a randomized, open-label, multicenter Phase III clinical trial evaluating the efficacy and safety of Dato-DXd compared to standard chemotherapy in patients with BC. The primary endpoints were PFS assessed by an independent, blinded central review committee (BICR) according to the Response Evaluation Criteria in Solid Tumors (RECIST) v1.1, and OS. Secondary endpoints included, among others, investigator-assessed PFS, ORR, CR, and PR. In addition, the safety and tolerability of the therapy were evaluated.

The study enrolled 732 patients with unresectable or metastatic HR+/HER2- BC who had experienced disease progression following prior systemic therapy. Participants were randomly assigned to two groups: the treatment group receiving Dato-DXd at a dose of 6 mg/kg intravenously every 3 weeks (n=365) and a control group receiving Investigator’s Choice Chemotherapy (ICC) based on a single agent from among eribulin, capecitabine, vinorelbine, and gemcitabine at standard chemotherapy doses (n=367) (22, 23).

Preliminary results of the study, with a median follow-up of 10.8 months, showed that treatment with Dato DXd was associated with a 37% reduction in the risk of disease progression or death compared with the ICC arm (HR 0.63; P < 0.0001). The median PFS was 6.9 months in the study group and 4.9 months in the control group; accordingly, the ORR was 36.4% and 22.9%. DCR was assessed at 12 weeks and was 75.3% in the Dato-DXd group and 63.8% in the ICC group. Additionally, the median DoR was 6.7 months and 5.7 months, respectively. The incidence of TRAEs of any grade was slightly higher in the study group at 93.6%, compared to 86.3% in the ICC group. In contrast, TRAEs of grade 3 or higher were observed in a significantly lower percentage of patients in the Dato-DXd group (20.8% vs. 44.7%). The most commonly reported adverse events were nausea, stomatitis, alopecia, fatigue, dry eyes, vomiting, constipation, and keratitis (23).

The final analysis of the study, conducted after a median follow-up of 22.8 months, confirmed the therapeutic benefits of Dato-DXd. It demonstrated the superiority of Dato-DXd in terms of PFS as assessed by BICR, with an HR of 0.63 (a 37% reduction in the risk of progression or death), a median PFS of 6.9 vs. 4.9 months, an ORR of 36.4% vs. 22.9%, and a DCR at 12 weeks of 75.3% vs. 63.8% for Dato-DXd and ICC, respectively. No significant difference was observed for OS (HR 1.01; median 18.6 vs. 18.3 months), which may have resulted from subsequent treatment, including ADCs, which were used more frequently in the ICC arm (24%) than in the Dato-DXd group (12.3%). A *post hoc* analysis adjusting for the impact of subsequent therapies with other ADCs (SG, T-DXd, or SG+T-DXd) suggested a numerical improvement in OS for Dato-DXd (median 19.1 vs. 17.5 months; HR 0.86), highlighting the drug’s potential long-term benefit with minimal influence from subsequent therapies. From an immunological perspective, this indicates that the spatial and molecular modifications induced by prior Dato-DXd exposure do not precipitate absolute cross-resistance to subsequent anti-Trop-2 or anti-HER2 constructs, supporting a model of persistent microenvironmental vulnerability. The individual efficacy parameters evaluated and their comparative results are detailed in **Table 4** (22).

**Table 4:** Final results of the TROPION-Breast01 study (22).

Variable	Dato-DXD (n=365)	ICC (n=367)
Median OS	18.6 months	18.3 months
ORR	36.7%	22.1%
CR	0.8%	0%
PR	35.9%	22.1%
SD	47.4%	48.8%
DCR at 12 weeks	80.8%	66.8%
Median DoR	7.2 months	6.0 months
PFS	6.9 months	4.9 months
PFS2	11.7 months	10.4 months

Long-term follow-up of patients also confirmed the safety of the therapy. TRAEs were observed in 94.7% of the study group and in 86.3% of the control group. In contrast, the incidence of Grade 3 or higher TRAEs was 22.2% and 45.6%, respectively. The most common TRAEs were nausea (51.9% vs. 23.6%, with Grade 3 or higher at 1.4% vs. 0.6%), stomatitis (51.4% vs. 23.6%, with grade 3 or higher at 1.4% vs. 0.6%), and fatigue (25% vs. 18.2%, with 1.9% vs. 2%). Hematologic disorders were observed significantly more frequently in patients receiving ICC. Neutropenia was observed in 11.7% vs. 43.3%, with 1.1% vs. 31.1% being grade 3 or higher; leukopenia in 7.5% vs. 17.4%, with 0.6% vs. 7.4% being grade 3 or higher; and anemia in 12.2% vs. 19.9%, with grade ≥3 anemia in 1.4% vs. 2.6% (22).

### 4.3 Summary of study results

Across the evaluated clinical trials, Dato-DXd demonstrated robust and highly consistent antitumor activity, with ORRs ranging from 26.8% to 36.4% and a median PFS extending from 6.9 to 8.3 months. Enhanced objective response rates in the Phase III setting highlight the efficacy of optimized dosing strategies. The drug’s safety profile remained predictable across trials, characterized predominantly by low-grade mucosal and ocular toxicities rather than systemic myelosuppression, thereby preserving systemic immune competence. **Table 5** presents the aggregated results of available and eligible clinical trials on Dato-DXd therapy in patients with HR+/HER2- BC and TNBC.

**Table 5:** Summary of studies included in the review. NE, not reached/not estimable.

Study	TROPION-PanTumor01	TROPION-Breast01
Population	85	732
Intervention	Dato-DXd	Dato-DXd
Type of examination	Single-arm	RCT
Year of study	2024	2024
TRAEs ≥G3	HR+/HER2– BC 41.5% TNBC 52.3%	22.2%
ORR	HR+/HER2– BC 26.8% TNBC 31.8%	36.7%
Median PFS	HR+/HER2– BC 8.3 months TNBC 4.4 months	6.9 months
Median DoR	HR+/HER2– BC NE TNBC 16.8 months	7.2 months
Median OS	HR+/HER2- BC NE TNBC 13.5 months	18.6 months
CR	HR+/HER2– BC 0% TNBC 2.3%	0.8%
PR	HR+/HER2– BC 26.8% TNBC 29.5%	35.9%
SD	HR+/HER2– BC 56.1% TNBC 40.9%	47.4%
PD	HR+/HER2– BC 12.2% TNBC 18.2%	13.7%
Citation	(22)	(23,24)

## 5 Discussion

This systematic review reflects the early stage of research on Dato-DXd for the treatment of BC, for which the number of available clinical trials remains limited. Despite this limitation, the studies included in the analysis provide key clinical data, including one Phase III RCT (TROPION-Breast01) and an early Phase I trial (TROPION-PanTumor01), allowing for a preliminary assessment of the therapy’s efficacy and safety.

Advanced HR+/HER2– BC and TNBC remain therapeutic challenges, particularly in patients with disease progression following standard systemic therapy. In populations previously treated with hormone therapy, chemotherapy, or other treatment regimens, the efficacy of conventional cytotoxic drugs is limited, and subsequent lines of therapy are associated with increasing toxicity and a deteriorating general condition of patients. In the case of TNBC, an additional challenge is the absence of hormone receptors and HER2, which precludes the use of targeted therapies, leading to the most unfavorable prognosis among all BC subtypes. Consequently, there is a growing need for new therapeutic strategies that combine high anticancer efficacy with a predictable and acceptable safety profile, minimizing the burden of adverse effects on the patient. In this context, particular attention is being paid to the broad therapeutic potential of ADCs.

Trop-2 is commonly overexpressed in HR+/HER2- and TNBC tumors, making it an attractive therapeutic target in BC oncology. Currently, SG and Dato-DXd represent the frontline of Trop-2-targeted ADCs, while trastuzumab deruxtecan (T-DXd) targets HER2 but shares the same topoisomerase I inhibitor payload (DXd). T-DXd was approved in December 2019 for the treatment of patients with unresectable or metastatic HER2+ BC, while SG was approved in April 2020 for the treatment of mTNBC (24, 25). Dato-DXd is the newest of these drugs, having received approval in January 2025 for the treatment of patients with unresectable or metastatic HR+/HER2- subtype BC following prior hormone therapy and chemotherapy (20).

Dato-DXd stands out from other ADCs due to its lower antibody-to-payload ratio (DAR≈4), which translates to a more predictable and controlled toxicity profile, consisting mainly of TRAEs related to mucosal membranes, gastrointestinal disturbances, and hematologic abnormalities. While the classic mechanism of action involves Trop-2 binding, rapid internalization, and intracellular payload release, its clinical potency is significantly augmented by a robust bystander killing effect. By successfully eradicating neighboring tumor cells irrespective of baseline Trop-2 density, this bystander activity serves as a critical mechanism to overcome intratumoral heterogeneity, which is a major driver of therapeutic resistance in advanced BC (26). From an immunological perspective, preclinical models suggest that this bystander efficacy may dynamically alter the TME. It is hypothesized that the lipophilic DXd payload freely diffuses across cell membranes, eliminating not only heterogeneous Trop-2-negative tumor subclones but also adjacent immunosuppressive cells and tumor-associated stromal elements. This localized destruction is thought to disrupt the protective architecture of the tumor niche, reducing microenvironmental immune evasion. Concurrently, this stromal deconstruction and the alleviation of physical barriers facilitate a more efficient trafficking of adaptive immune cells into the core of the tumor, creating an ideal substrate for combined immunotherapeutic interventions.

Analysis of the clinical data yields highly encouraging outcomes across both HR+/HER2– and TNBC cohorts. However, it is crucial to emphasize the different levels of clinical evidence supporting these indications. For HR+/HER2- disease, efficacy is supported by randomized Phase III data (TROPION-Breast01), whereas evidence for TNBC currently relies on early-phase, single-arm results (TROPION-PanTumor01). In the Phase I TROPION-PanTumor01 trial, Dato-DXd demonstrated robust efficacy; patients with HR+/HER2– BC achieved a median PFS of 8.3 months, whereas the median PFS in the aggressive TNBC cohort was documented at 4.4 months. Conversely, the median OS was documented at 13.5 months in TNBC patients and was not reached in the HR+/HER2– arm. Regarding safety, the most frequent TRAEs comprised mild-to-moderate events including stomatitis, nausea, and fatigue. This toxicity profile was deemed clinically acceptable, as the observed adverse effects proved predictable, manageable, and consistent with the established mechanism of the drug’s payload.

However, complex resistance mechanisms may inevitably restrict the long-term efficacy of Dato-DXd. In patients extensively pretreated with alternative topoisomerase I-targeted ADCs, specific genomic alterations within the TOP1 gene (e.g., S57C, R364H, W401C, and G359E) can emerge, mediating cross-resistance and markedly reducing the therapeutic benefit of subsequent ADC lines. Additionally, adaptive modifications in DNA damage response (DDR) pathways coupled with comprehensive tumor microenvironment remodeling can physically impede optimal drug penetration and promote tumor cell survival, highlighting a critical clinical challenge that necessitates targeted counter-strategies (27). Particularly, activation of the CAF-FAK signaling axis often leads to dense extracellular matrix deposition, forming a structural barrier against ADC macromolecule delivery.

In the Phase III TROPION Breast01 trial, Dato DXd demonstrated significant and clinically meaningful advantage over ICC in terms of PFS (median 6.9 vs. 4.9 months; HR 0.63) and ORR (36.4% vs 22.9%). The lack of a significant difference in OS (18.6 vs 18.3 months; HR 1.01) can be largely attributed to the confounding impact of subsequent lines of cross-over therapies, including other ADCs, which were used more frequently in the ICC arm; in a post-hoc analysis adjusted for these subsequent therapies, this suggested a numerical improvement in OS for Dato DXd (median 19.1 vs. 17.5 months; HR 0.86). The toxicity profile of Dato DXd remained favorable: grade ≥3 adverse events occurred less frequently than in the ICC group (22.2% vs 45.6%), low-grade TRAEs predominated, and severe hematotoxicity was markedly reduced.

When comparing the efficacy of available ADCs, it is crucial to contextualize the Dato-DXd study results within the established data for other ADCs, while accounting for differences in study populations and prior treatment intensity. The Phase III ASCENT trial, which evaluated the efficacy of SG in patients with mTNBC, demonstrated a significant improvement in clinical outcomes compared to patients receiving chemotherapy. The median PFS in the SG arm was 4.8 months, compared to 1.7 months in patients receiving chemotherapy. In contrast, median OS was 12.1 and 6.7 months, respectively (28). In turn, in the DESTINY-Breast04 trial evaluating T-DXd in patients with HER2-low BC, significant improvements in both PFS and OS were demonstrated. The median PFS was 8.8 months in the T-DXd arm compared to 4.2 months for chemotherapy, while the median OS reached 22.9 months vs. 16.8 months (29). Although cross-trial comparisons demand strict caution due to distinct baseline demographic variables and sequential therapy configurations, the 6.9-month median PFS achieved in TROPION-Breast01 establishes Dato-DXd as an exceptionally robust and highly competitive salvage option, uniquely optimizing the efficacy-to-toxicity ratio in the advanced setting.

Ultimately, while caution is warranted when interpreting these multi-trial data, differences in study populations, prior treatment intensities, and clinical trial designs fundamentally preclude direct cross-trial efficacy comparisons between Dato-DXd and other approved ADCs like sacituzumab govitecan (SG) or trastuzumab deruxtecan (T-DXd). In this context, Dato-DXd appears to occupy a complementary rather than mutually exclusive role in the metastatic treatment sequence. This clinical reality underscores the critical importance of future head-to-head trials and biomarker-driven longitudinal analyses to determine the optimal strategic sequencing of these potent agents.

To maximize the therapeutic paradigm of Dato-DXd, clinical focus is rapidly transitioning toward shifting this agent into earlier lines of management and exploring innovative combination strategies, which take on particular significance in the ongoing Phase III programs (e.g., TROPION-Breast02 and TROPION-Breast03). Of profound immunological interest is the evaluation of combinations with immune checkpoint inhibitors, such as durvalumab in the TROPION-Breast04 study. Mechanistically, preclinical evidence indicates that this synergy may rely heavily on the cytotoxic release of damage-associated molecular patterns (DAMPs) and subsequent immunogenic cell death (ICD) induced by the DXd payload. This coordinated process has the theoretical potential to convert an immunologically ‘cold’, immunosuppressed tumor microenvironment into an immune-reactive ‘hot’ niche by a robust recruitment and infiltration of tumor-infiltrating lymphocytes (TILs), particularly cytotoxic CD8+ T cells. This inflamed phenotype ultimately optimizes the therapeutic window for anti-PD-1/PD-L1 mediated T-cell reactivation and drives long-term durable responses (30, 31).

Despite these encouraging clinical signals, the available data synthesized in this review possess several structural limitations. First, early-phase studies involved relatively small cohorts of patients, which limits the generalizability of the results, while the Phase III TROPION-Breast01 trial did not demonstrate a statistically significant improvement in overall survival during its initial analysis—a finding potentially confounded by the impact of subsequent lines of cross-over treatments. Additionally, the heterogeneity of Trop-2 expression across different BC subtypes, coupled with the molecular complexity of potential cross-resistance between topoisomerase I inhibitor-based conjugates, continues to complicate the precise delineation of the optimal target population and therapeutic sequence.

Concurrently, innovative strategies to optimize treatment safety are actively being developed. In clinical trials such as TROPION-DM, proactive prophylactic interventions are being evaluated, including the utilize of steroid-based mouthwashes containing dexamethasone, aimed at reducing the frequency and severity of oral mucositis, which remains one of the most common adverse effects associated with Dato-DXd treatment. These supportive approaches may significantly improve overall treatment tolerance, preserve dose intensity, and enable longer, more effective use of this therapy in routine clinical practice (32). At the same time, generating robust real-world evidence (RWE) data will be essential to assess Dato-DXd’s efficacy and safety in more heterogeneous patient populations, bridging the gap between highly controlled clinical trials and everyday oncology practice.

In summary, Dato-DXd represents a significant therapeutic advance, supported by robust Phase III randomized evidence in advanced HR+/HER2– BC, and demonstrating promising early clinical activity based on Phase I data in TNBC. Its favorable safety profile, robust activity in previously heavily pretreated populations, and mechanism of action that successfully overcomes tumor heterogeneity make it a pivotal component of contemporary therapeutic strategies. Further research focused on predictive biomarkers, resistance mechanisms, and optimal treatment sequences and combinations will be crucial for fully realizing the potential of this class of drugs in BC oncology. Additionally, this systematic review should be interpreted as an analysis of current, early clinical data (emerging evidence), which forms the foundational basis for further research into the optimal utilization of Dato-DXd. Ultimately, its ability to successfully target tumor vulnerabilities while offering a scaffold for combination regimens highlights its defining role in the next generation of personalized cancer treatment.

## 6 Limitations

In this review, several factors should be considered when interpreting the results. The main limitation of the review is the small number of completed clinical trials available, due to the early stage of development of the therapy under analysis. The current evidence is based solely on the results of two clinical trials, including only one RCT, which limits the comprehensiveness and strength of the conclusions. Data from the Phase I trial are exploratory in nature and come from a single-arm analysis, precluding a direct comparison of Dato-DXd’s efficacy with other therapeutic options. Furthermore, these studies included a highly selected patient population, primarily in good general health and without significant comorbidities, which limits the ability to generalize the results to broader clinical populations. From a statistical perspective, this highly selective inclusion criterion inherently restricts the external validity of the clinical findings, underrepresenting patients with lower performance statuses or significant organic dysfunctions who frequently present in real-world oncology clinics.

A significant limitation remains the lack of direct comparisons with other modern therapies, which prevents determining the place of this treatment strategy in current clinical management algorithms. Differences in population characteristics, treatment lines, and the endpoints used introduce heterogeneity, limiting the consistency and interpretation of the results. This methodological divergence is particularly evident when contrasting cross-trial baseline characteristics, where prior exposure to topoisomerase I inhibitors, specific lines of chemotherapy, or immune checkpoint inhibitors creates an unmeasurable chronological and immunological bias, confounding any indirect comparative efficacy analysis and leaving the impact of prior immune-mediated microenvironmental remodeling on Dato-DXd resistance largely unquantified.

Available data on long-term outcomes, including overall survival and response durability, remain limited. Not all relevant endpoints, such as patients’ quality of life, were consistently reported. The limited maturity of some of the analyses may affect the stability of the results, which may change with further follow-up. Consequently, the current quantitative estimates for absolute HR and PFS curves must be interpreted with caution, as immature data structures are mathematically prone to overestimating early treatment benefits before reaching a true statistical plateau. Furthermore, a critical translational limitation is the current lack of validated, immune-centric or molecular biomarkers beyond raw Trop-2 expression, which limits the ability to predict which patients will develop early resistance versus those who will achieve long-term durability of response.

Despite these limitations, the review was conducted in accordance with the PRISMA guidelines, ensuring a transparent and reproducible process for study selection and analysis. This analysis presents the current state of knowledge regarding Dato-DXd in BC therapy and highlights the need for further research, including randomized and RWE studies, to obtain a more comprehensive assessment of the efficacy and safety of this therapy. Integrating such prospective data will be paramount to bridge the current translational gap, ensuring that clinical efficacy profiles observed in highly controlled trial settings successfully translate into durable, long-term therapeutic effectiveness within unselected, heterogeneous breast cancer cohorts.

## 7 Conclusion

Advanced, metastatic, and inoperable BC remains a significant therapeutic challenge, particularly following the failure of standard treatment. In this context, Dato-DXd represents a new direction in Trop-2-targeted therapy, offering a proven survival benefit in HR+/HER2- patients and showing early promise in TNBC. The pharmacological profile of Dato-DXd, including the “bystander” effect, explains its activity in heterogeneous tumors, and its favorable efficacy-to-toxicity ratio allows for longer maintenance of therapy. However, its actual advantage over other ADCs remains unclear, and determining the optimal point in the treatment sequence and identifying patients who will benefit most will be of key importance. From a mechanistic standpoint, preclinical studies propose that leveraging this bystander activity may allow for the systematic eradication of surrounding antigen-negative cells, effectively reshaping the cellular composition of the TME and counteracting clonal selection pathways that typically drive early therapeutic failure.

The available clinical data indicate a clear and clinically significant improvement in PFS and a higher ORR compared with standard chemotherapy, with a more favorable safety profile regarding severe TRAEs, despite frequent low-grade events typical of this class of drugs. The lack of a clear improvement in OS should be considered in the context of the impact of subsequent lines of treatment, which may have significantly modified the observed results. This profound crossover effect underscores the necessity of implementing rigorous multi-state models in future clinical trial designs to accurately censor subsequent therapeutic intervention biases and isolate the true survival benefit of first-line ADC exposure.

Dato-DXd represents a favorable therapeutic option and a potentially complementary therapy in the treatment sequence for selected populations of BC patients. Ongoing clinical trials will provide more comprehensive data on the efficacy and safety of this therapy in the future. Further studies should focus not only on optimizing treatment sequences and identifying predictive biomarkers, but also on evaluating combination therapy strategies, including in combination with immunotherapy, which may further enhance treatment efficacy and overcome resistance mechanisms. By strategically pairing the cytotoxic payload delivery with immune checkpoint inhibitors, future regimens aim to exploit ICD cascades. While currently based on biological rationale and preclinical models, it is hoped that this approach may help turn immunologically quiescent breast tumors into highly active niches responsive to sustained T-cell mediated anti-tumor immunity.
